# Poly[di-μ_2_-azido-μ_3_-pyrazine-2-carboxyl­ato-cadmium(II)]

**DOI:** 10.1107/S1600536809012227

**Published:** 2009-04-10

**Authors:** Cai-Yun Li, Pei-Fan Li, Hui-Min Jin

**Affiliations:** a Department of Pharmaceutical Science, Tianjin Medical College, Tianjin 300222, People’s Republic of China

## Abstract

The title compound, [Cd(C_5_H_3_N_2_O_2_)(N_3_)]_*n*_, has been pre­pared by the reaction of pyrazine-2-carboxylic acid, cadmium(II) nitrate and sodium azide. In the structure, the Cd^II^ atom is six-coordinated by two azide anions and three pyrazine-2-carboxyl­ate ligands. Each pyrazine-2-carboxyl­ate ligand bridges three Cd^II^ atoms, whereas the azide ligand bridges two Cd^II^ atoms, resulting in the formation of a two-dimensional metal–organic polymer developing parallel to the (100) plane.

## Related literature

For metal–azide complexes, see: Mondal & Mukherjee (2008 ▶); Gu *et al.* (2007 ▶); Monfort *et al.* (2000 ▶). For the coordination modes of the azide anion, see: Shen *et al.* (2000 ▶). For metal–azide complexes with charged ligands, see: Escuer *et al.* (1997 ▶). For the synthesis of high-dimensional azide compounds with negatively charged ligands, see: Liu *et al.* (2005 ▶).
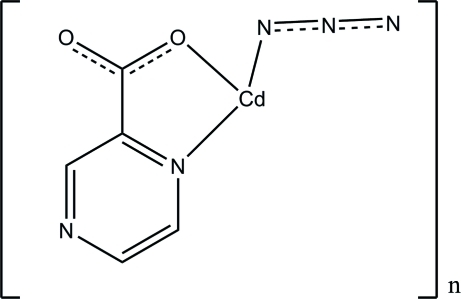


## Experimental

### 

#### Crystal data

[Cd(C_5_H_3_N_2_O_2_)(N_3_)]
*M* *_r_* = 277.52
Monoclinic, 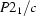

*a* = 11.857 (2) Å
*b* = 9.839 (2) Å
*c* = 6.6250 (13) Å
β = 100.33 (3)°
*V* = 760.4 (3) Å^3^
*Z* = 4
Mo *K*α radiation
μ = 2.84 mm^−1^
*T* = 293 K
0.20 × 0.18 × 0.15 mm

#### Data collection

Rigaku SCXmini diffractometer
Absorption correction: multi-scan (*ABSCOR*; Higashi, 1995 ▶) *T*_min_ = 0.537, *T*_max_ = 0.643
7718 measured reflections
1741 independent reflections
1517 reflections with *I* > 2σ(*I*)
*R*_int_ = 0.067

#### Refinement

*R*[*F* ^2^ > 2σ(*F* ^2^)] = 0.046
*wR*(*F* ^2^) = 0.117
*S* = 1.20
1741 reflections
118 parameters
H-atom parameters constrained
Δρ_max_ = 0.74 e Å^−3^
Δρ_min_ = −1.09 e Å^−3^

### 

Data collection: *SCXmini* (Rigaku, 2006 ▶); cell refinement: *PROCESS-AUTO* (Rigaku, 1998 ▶); data reduction: *PROCESS-AUTO*; program(s) used to solve structure: *SHELXS97* (Sheldrick, 2008 ▶); program(s) used to refine structure: *SHELXL97* (Sheldrick, 2008 ▶); molecular graphics: *ORTEPIII* (Burnett & Johnson, 1996 ▶) and *PLATON* (Spek, 2009 ▶); software used to prepare material for publication: *SHELXL97*.

## Supplementary Material

Crystal structure: contains datablocks global, I. DOI: 10.1107/S1600536809012227/dn2435sup1.cif

Structure factors: contains datablocks I. DOI: 10.1107/S1600536809012227/dn2435Isup2.hkl

Additional supplementary materials:  crystallographic information; 3D view; checkCIF report

## Figures and Tables

**Fig. 1.**
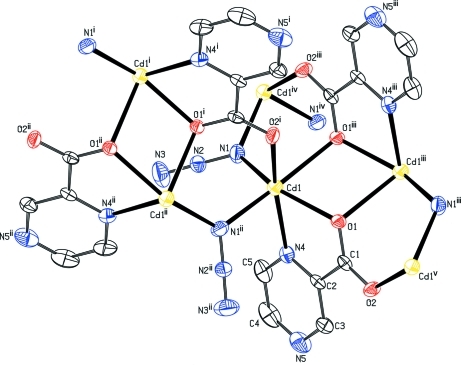
A view of the title compound showing the coordination of Cd atom with the atom-labelling scheme. Ellipsoids are drawn at the 50% probability level. H atom have been omitted for clarity. [Symmetry codes: (i) -x+1, y-1/2, -z+1/2; (ii) x, -y+1/2, z-1/2; (iii) -x+1, -y+1, -z+1; (iv) x, -y+1/2, z+1/2; (v) -x+1, y+1/2, -z+1/2]

## supplementary crystallographic information

### Comment

Recently, metal azide complexes have attracted great attention.(Mondal & Mukherjee, 2008; Gu *et al.*, 2007). The azide anion have rich coordinated modes. (Shen,*et al.*, 2000). In this sense, lots metal-azide complexes have been reported.(Monfort,*et al.*, 2000). In most of the compounds reported to date, the coligands are neutral organic ligands, while charged ligands are very scarce (Escuer *et al.*, 1997). Synthesizing high-dimensional compounds with azide and negatively charged ligands represents then a challenge for researchers working in this field. (Liu *et al.*, 2005)

In the title compound, the cadmium atom is six coordinated by two azide anions and three pyrazine-2-carboxylate (Fig. 1). Each pyrazine-2-carboxylate bridges three cadmium atoms whereas the azide is bridging two cadmium atoms resulting in the formation of a two dimensional metal organic polymer developping parallel to the (1 0 0) plane.

### Experimental

A mixture of cadmium(II)nitrate and sodium azide (1 mmol), pyrazine-2-carboxylate acid(0.5 mmol), in 10 ml of water was sealed in a Teflon-lined stainless-steel Parr bomb that was heated at 363 K for 48 h. Pink crystals of the title complex were collected after the bomb was allowed to cool to room temperature.Yield 30% based on cadmium(II). Caution:Metal azides may be explosive. Although we have met no problems in this work, only a small amount of them should be prepared and handled with great caution.

### Refinement

Hydrogen atoms were included in calculated positions and treated as riding on their parent C atoms with C—H = 0.93Å and *U*_iso_(H) = 1.2*U*_eq_(C).

### Crystal data

**Table:**

[Cd(C_5_H_3_N_2_O_2_)(N_3_)]	*F*(000) = 528
*M**_r_* = 277.52	*D*_x_ = 2.424 Mg m^−^^3^
Monoclinic, *P*2_1_/*c*	Mo *K*α radiation, λ = 0.71073 Å
Hall symbol: -P 2ybc	Cell parameters from 7230 reflections
*a* = 11.857 (2) Å	θ = 3.1–27.5°
*b* = 9.839 (2) Å	µ = 2.84 mm^−^^1^
*c* = 6.6250 (13) Å	*T* = 293 K
β = 100.33 (3)°	Block, yellow
*V* = 760.4 (3) Å^3^	0.2 × 0.18 × 0.15 mm
*Z* = 4	

### Data collection

**Table:**

Rigaku SCXmini diffractometer	1741 independent reflections
Radiation source: fine-focus sealed tube	1517 reflections with *I* > 2σ(*I*)
graphite	*R*_int_ = 0.067
ω scans	θ_max_ = 27.5°, θ_min_ = 3.5°
Absorption correction: multi-scan (*ABSCOR*; Higashi, 1995)	*h* = −15→15
*T*_min_ = 0.537, *T*_max_ = 0.643	*k* = −12→12
7718 measured reflections	*l* = −8→8

### Refinement

**Table:**

Refinement on *F*^2^	Primary atom site location: structure-invariant direct methods
Least-squares matrix: full	Secondary atom site location: difference Fourier map
*R*[*F*^2^ > 2σ(*F*^2^)] = 0.046	Hydrogen site location: inferred from neighbouring sites
*wR*(*F*^2^) = 0.117	H-atom parameters constrained
*S* = 1.20	*w* = 1/[σ^2^(*F*_o_^2^) + (0.0408*P*)^2^ + 2.6851*P*] where *P* = (*F*_o_^2^ + 2*F*_c_^2^)/3
1741 reflections	(Δ/σ)_max_ < 0.001
118 parameters	Δρ_max_ = 0.74 e Å^−^^3^
0 restraints	Δρ_min_ = −1.09 e Å^−^^3^

### Special details

**Table:**

Geometry. All esds (except the esd in the dihedral angle between two l.s. planes) are estimated using the full covariance matrix. The cell esds are taken into account individually in the estimation of esds in distances, angles and torsion angles; correlations between esds in cell parameters are only used when they are defined by crystal symmetry. An approximate (isotropic) treatment of cell esds is used for estimating esds involving l.s. planes.
Refinement. Refinement of *F*^2^ against ALL reflections. The weighted *R*-factor *wR* and goodness of fit *S* are based on *F*^2^, conventional *R*-factors *R* are based on *F*, with *F* set to zero for negative *F*^2^. The threshold expression of *F*^2^ > σ(*F*^2^) is used only for calculating *R*-factors(gt) *etc*. and is not relevant to the choice of reflections for refinement. *R*-factors based on *F*^2^ are statistically about twice as large as those based on *F*, and *R*- factors based on ALL data will be even larger.

### Fractional atomic coordinates and isotropic or equivalent isotropic displacement parameters (Å^2^)

**Table:**

	*x*	*y*	*z*	*U*_iso_*/*U*_eq_	
Cd1	0.42263 (4)	0.36045 (4)	0.34194 (7)	0.02481 (18)	
N1	0.3555 (5)	0.1927 (5)	0.5059 (8)	0.0287 (12)	
N2	0.2937 (5)	0.1135 (5)	0.4006 (8)	0.0248 (12)	
N3	0.2327 (6)	0.0399 (7)	0.3035 (10)	0.0466 (17)	
N4	0.2558 (4)	0.4910 (5)	0.3100 (7)	0.0231 (11)	
N5	0.0689 (5)	0.6646 (7)	0.1984 (10)	0.0390 (15)	
O1	0.4718 (3)	0.5816 (4)	0.3145 (6)	0.0197 (8)	
O2	0.4043 (4)	0.7823 (4)	0.1835 (6)	0.0250 (9)	
C1	0.3913 (5)	0.6650 (6)	0.2480 (8)	0.0198 (12)	
C2	0.2694 (5)	0.6193 (6)	0.2517 (8)	0.0194 (12)	
C3	0.1769 (5)	0.7052 (7)	0.1982 (9)	0.0277 (14)	
H3A	0.1898	0.7942	0.1609	0.033*	
C4	0.0571 (6)	0.5372 (9)	0.2550 (11)	0.0422 (19)	
H4A	−0.0162	0.5044	0.2575	0.051*	
C5	0.1493 (6)	0.4509 (8)	0.3106 (10)	0.0327 (16)	
H5B	0.1362	0.3623	0.3494	0.039*	

### Atomic displacement parameters (Å^2^)

**Table:**

	*U*^11^	*U*^22^	*U*^33^	*U*^12^	*U*^13^	*U*^23^
Cd1	0.0233 (3)	0.0213 (3)	0.0287 (3)	−0.00024 (18)	0.00174 (19)	0.00118 (18)
N1	0.038 (3)	0.023 (3)	0.024 (3)	−0.008 (2)	0.002 (2)	−0.001 (2)
N2	0.022 (3)	0.025 (3)	0.027 (3)	−0.003 (2)	0.003 (2)	0.000 (2)
N3	0.051 (4)	0.047 (4)	0.040 (3)	−0.023 (3)	0.001 (3)	−0.004 (3)
N4	0.022 (3)	0.025 (3)	0.023 (2)	−0.004 (2)	0.005 (2)	−0.003 (2)
N5	0.018 (3)	0.054 (4)	0.044 (3)	0.002 (3)	0.004 (3)	−0.008 (3)
O1	0.014 (2)	0.020 (2)	0.025 (2)	−0.0004 (16)	0.0012 (16)	0.0018 (17)
O2	0.023 (2)	0.018 (2)	0.033 (2)	−0.0007 (17)	0.0035 (18)	0.0075 (18)
C1	0.018 (3)	0.026 (3)	0.013 (3)	−0.004 (2)	0.000 (2)	−0.001 (2)
C2	0.015 (3)	0.028 (3)	0.014 (2)	−0.001 (2)	−0.001 (2)	−0.002 (2)
C3	0.019 (3)	0.031 (4)	0.031 (3)	0.000 (3)	−0.001 (3)	−0.001 (3)
C4	0.018 (4)	0.071 (6)	0.040 (4)	−0.012 (3)	0.011 (3)	−0.010 (4)
C5	0.022 (3)	0.043 (4)	0.034 (4)	−0.016 (3)	0.010 (3)	−0.003 (3)

### Geometric parameters (Å, °)

**Table:**

Cd1—N1	2.202 (5)	N5—C4	1.324 (10)
Cd1—O2^i^	2.226 (4)	N5—C3	1.342 (8)
Cd1—O1	2.268 (4)	O1—C1	1.275 (7)
Cd1—N4	2.336 (5)	O2—C1	1.250 (7)
Cd1—O1^ii^	2.460 (4)	C1—C2	1.517 (8)
N1—N2	1.203 (7)	C2—C3	1.380 (8)
N1—Cd1^iii^	2.286 (5)	C3—H3A	0.9300
N2—N3	1.138 (8)	C4—C5	1.381 (11)
N4—C5	1.324 (8)	C4—H4A	0.9300
N4—C2	1.339 (8)	C5—H5B	0.9300
			
N1—Cd1—O2^i^	101.42 (19)	C2—N4—Cd1	113.6 (4)
N1—Cd1—O1	151.56 (18)	C4—N5—C3	115.6 (6)
O2^i^—Cd1—O1	94.12 (15)	C1—O1—Cd1	117.2 (4)
N1—Cd1—N1^iv^	102.45 (15)	C1—O1—Cd1^ii^	113.3 (3)
O2^i^—Cd1—N1^iv^	90.68 (18)	Cd1—O1—Cd1^ii^	104.11 (15)
O1—Cd1—N1^iv^	101.01 (17)	C1—O2—Cd1^v^	121.1 (4)
N1—Cd1—N4	94.7 (2)	O2—C1—O1	125.6 (5)
O2^i^—Cd1—N4	163.79 (17)	O2—C1—C2	117.0 (5)
O1—Cd1—N4	71.99 (16)	O1—C1—C2	117.4 (5)
N1^iv^—Cd1—N4	84.05 (18)	N4—C2—C3	121.3 (6)
N1—Cd1—O1^ii^	83.47 (16)	N4—C2—C1	116.7 (5)
O2^i^—Cd1—O1^ii^	80.03 (14)	C3—C2—C1	122.0 (5)
O1—Cd1—O1^ii^	75.89 (15)	N5—C3—C2	122.2 (6)
N1^iv^—Cd1—O1^ii^	169.88 (17)	N5—C3—H3A	118.9
N4—Cd1—O1^ii^	103.82 (15)	C2—C3—H3A	118.9
N2—N1—Cd1	115.8 (4)	N5—C4—C5	122.6 (6)
N2—N1—Cd1^iii^	119.1 (4)	N5—C4—H4A	118.7
Cd1—N1—Cd1^iii^	124.0 (2)	C5—C4—H4A	118.7
N3—N2—N1	178.0 (7)	N4—C5—C4	121.7 (7)
C5—N4—C2	116.6 (6)	N4—C5—H5B	119.1
C5—N4—Cd1	128.9 (5)	C4—C5—H5B	119.1

Symmetry codes: (i) −*x*+1, *y*−1/2, −*z*+1/2; (ii) −*x*+1, −*y*+1, −*z*+1; (iii) *x*, −*y*+1/2, *z*+1/2; (iv) *x*, −*y*+1/2, *z*−1/2; (v) −*x*+1, *y*+1/2, −*z*+1/2.
